# Light-Mediated Toxicity of Porphyrin-Like Pigments from a Marine Polychaeta

**DOI:** 10.3390/md18060302

**Published:** 2020-06-06

**Authors:** Mariaelena D’Ambrosio, Ana Catarina Santos, Alfonso Alejo-Armijo, A. Jorge Parola, Pedro M. Costa

**Affiliations:** 1 UCIBIO–Applied Molecular Biosciences Unit, Departamento de Ciências da Vida, Faculdade de Ciências e Tecnologia da Universidade Nova de Lisboa, 2829-516 Caparica, Portugal; acf.santos@campus.fct.unl.pt; 2 LAQV–Associate Laboratory for Green Chemistry, Departamento de Química, Faculdade de Ciências e Tecnologia da Universidade Nova de Lisboa, 2829-516 Caparica, Portugal; aaa00010@red.ujaen.es (A.A.-A.); ajp@fct.unl.pt (A.J.P.)

**Keywords:** porphyrinoids, annelida, marine bioproducts, HPLC-DAD, toxicity, photosensitizers

## Abstract

Porphyrins and derivatives form one of the most abundant classes of biochromes. They result from the breakdown of heme and have crucial physiological functions. Bilins are well-known representatives of this group that, besides significant antioxidant and anti-mutagenic properties, are also photosensitizers for photodynamic therapies. Recently, we demonstrated that the Polychaeta *Eulalia viridis*, common in the Portuguese rocky intertidal, holds a high variety of novel greenish and yellowish porphyrinoid pigments, stored as granules in the chromocytes of several organs. On the follow-up of this study, we chemically characterized pigment extracts from the worm’s skin and proboscis using HPLC and evaluated their light and dark toxicity in vivo and ex vivo using *Daphnia* and mussel gill tissue as models, respectively. The findings showed that the skin and proboscis have distinct patterns of hydrophilic or even amphiphilic porphyrinoids, with some substances in common. The combination of the two bioassays demonstrated that the extracts from the skin exert higher dark toxicity, whereas those from the proboscis rapidly exert light toxicity, then becoming exhausted. One particular yellow pigment that is highly abundant in the proboscis shows highly promising properties as a natural photosensitizer, revealing that porphyrinoids from marine invertebrates are important sources of these high-prized bioproducts.

## 1. Introduction

Biological pigments (biochromes) can be defined as any material from biological origin that results in color. They can have many functions, among which mimicking and communication are but a few, and are normally products of complex and varied biosynthetic pathways involving a wide span of enzymes [1]. Pigments resulting from these metabolic processes can be broadly divided in two classes: those that are directly responsible for organism colors and those that are colored secondary metabolites, which may or may not be directly involved with the organisms’ primary pigmentation [2]. One of the most abundant classes of natural pigments are tetrapyrroles, considered as the “pigments of life” [3] due to their role in photosynthesis, gas transport, and redox reactions. This class includes porphyrins, which are metabolites of heme.

Arguably, the best known porphyrinoids are bilins (also termed bilichromes or bile pigments) [2,4], whose coloration can vary between yellow, green, red, and brown [5]. They are secondary metabolites devoid of metal cores and are arranged in linear (chain) structures rather than in the customary cyclic configuration of porphyrins [2]. Biliverdin and bilirubin are notorious members of this group that, despite resulting from the breakdown of heme, hold important biological functions, such as antioxidants, in humans and other animals [6]. Like many other tetrapyrroles, bilins are photosensitive, which provides them with a high interest in photodynamic therapy (PDT), since they can generate singlet oxygen when irradiated, thus triggering localized cytotoxic effects [7]. In fact, depending on their structure, porphyrinoids have a distinctive absorption spectrum in the UV-visible region, characterized by a strong band around 420 nm, named the Soret band, and a series of low-intensity absorption bands at longer wavelengths, typically between 500 and 650 nm, termed Q bands (refer to Arnaut [8] for an overview). However, photoactivation may occur when the pigments are subjected to light from the whole visible spectrum [9]. Porphyrinoids may exert light-independent toxicity, nonetheless, which renders mandatory safeguarding a high light:dark toxicity ratio to uphold a compound’s value as photosensitizer [10]. Altogether, porphyrinoids are provided with a particular interest for biotechnological purposes as colorants, antimicrobial, biocides, or as biomarkers for the physiological status of animals [11,12,13].

Marine invertebrates, in particular, especially those of coastal environments, known for their bright and diversified coloration, seemingly have a wider span of porphyrinoids than their vertebrate counterparts. However, as for other natural products, the true diversity, nature, and function of these pigments in marine invertebrates remains largely unknown. Some of the first works on tetrapyrrole pigments from marine animals began with bonellin, a distinctive chlorin-like greenish pigment from the females of the Echiuran Polychaeta *Bonellia viridis* that is turned into a potent biocidal when photoactivated, protecting the animal from predators and biofoulants [14,15]. In turn, *Hediste* (*Nereis*) is a common intertidal worm that is known to have a diversified and seasonally changing pattern of pigments, in large part due to porphyrinoids resulting from endogenous heme breakdown [16].

Recently, we showed that an uncanny uniformly green Polychaeta, *Eulalia viridis*, an opportunistic predator of the rocky intertidal, owes its coloration to a multiplicity of endogenous porphyrin-like pigments whose abundance and distribution changes between organs [17,18,19,20]. These pigments, which seem to almost entirely replace common biochromes such as carotenoids and melanins, were found to be chiefly stored as granules allocated within unique specialized chromocytes [19,20]. The function and bio-reactivity of these pigments is not, however, fully understood. It was hypothesized that, more than mimicking the worm’s surroundings, these pigments offer important protection from sunlight and that, being porphyrinoids, they may also have toxic properties modulated by light. Additionally, *E. viridis* secretes toxins that, delivered by its copious mucus secretion, are used to immobilize and partly digest prey (mostly invertebrates, especially mussels, barnacles, and other Polychaeta) before extracting a portion of flesh via suction with its jawless but highly muscular proboscis [21]. This ability also led us to hypothesize a strong investment in chemical warfare on behalf of the species that may extend to its pigmentation. On the follow-up of our preceding research, the current work aimed at a comparative screening for potential light-mediated toxicity of the novel porphyrinoid pigments extracted from *E. viridis* skin and proboscis in view of its potential biotechnological value.

## 2. Results

### 2.1. Chemical Characterization of Pigments

**Figure 1:**
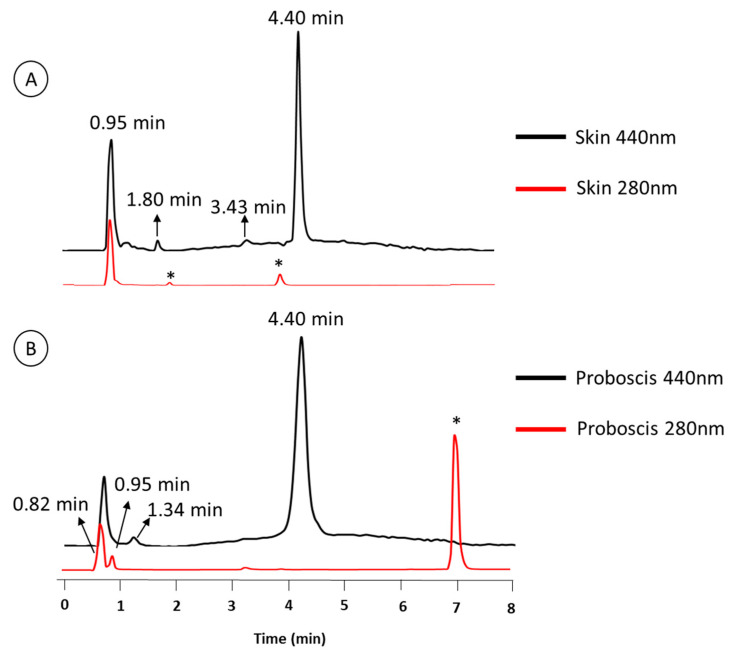
Representative HPLC-DAD profiles at 440 and 280 nm (black and red line, respectively). Pigments extracts from (**A**) skin, and (**B**) proboscis. Compounds with * did not present the characteristic absorption of porphyrin pigments and were not further addressed.

**Figure 2:**
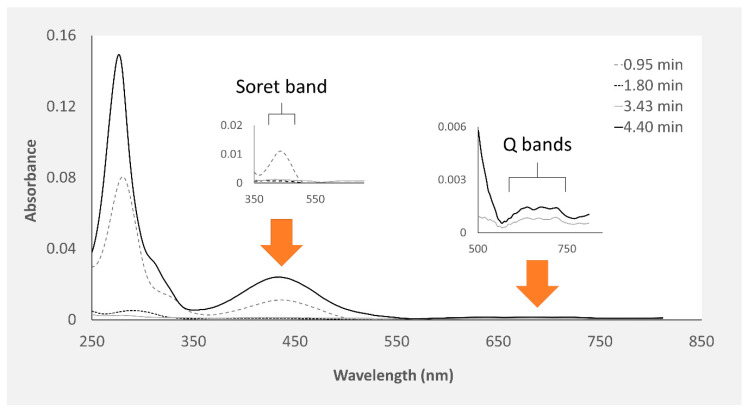
Absorption spectra of the four main pigments in *Eulalia viridis* skin crude pigment extracts spectra retrieved from HPLC-DAD analyses (each pigment’s spectrum is identified by its retention time). The Soret (350–500 nm) and Q (580–750 nm) bands typical of porphyrinoids are highlighted.

**Figure 3:**
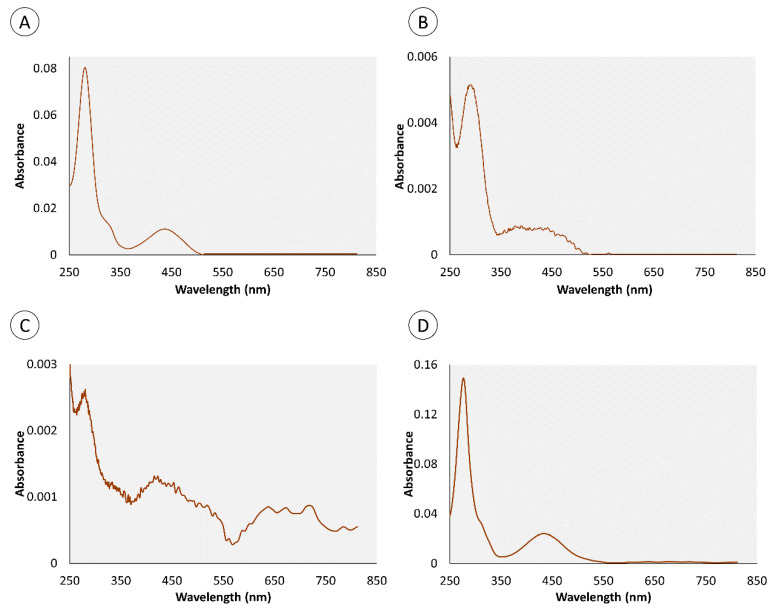
Spectra of the principal pigments in the crude extracts from the skin of *Eulalia viridis* fractionated by HPLC-DAD. The skin yielded mainly one greenish and three yellowish pigments. (**A**) yellow pigment (0.95 min); (**B**) yellow pigment (1.8 min); (**C**) green pigment (3.43 min); (**D**) yellow pigment (4.40 min). The pigments detected at retention times 1.80 and 3.34 min are specific to the skin.

In accordance with Martins et al. [20], the crude extracts in cold Dulbecco’s phosphate-buffered saline (PBS) from the proboscis and skin, being yellowish and greenish in color, respectively, were comprised of multiple pigments. Both extracts of skin and proboscis tissues were analyzed by HPLC-DAD and recorded at 280 and 440 nm (exemplified in Figure 1). For simplification purposes, this distinction based on visual inspection will be retained. Albeit variable among individual pigments, the absorption spectra of either extract had maxima within the UV range (ca. 280 nm), plus Soret bands between 350 and 500 nm and the characteristic Q bands (580–750 nm) of porphyrin-like pigments, which is particularly obvious for the main pigments fractionated from the skin (Figure 2). Among the main pigments in skin extracts that better fit the expected porphyrin signature, we found a yellowish pigment that was exclusive to this organ, with a retention time of 1.8 min. To this substance a greenish pigment was added with retention time 3.43 min and two yellow pigments with retention times 0.95 and 4.40 min (Figure 3). The latter, in particular, was found to be the most abundant pigment in skin extracts, as inferred from its higher absorbance (Figure 3D).

**Figure 4:**
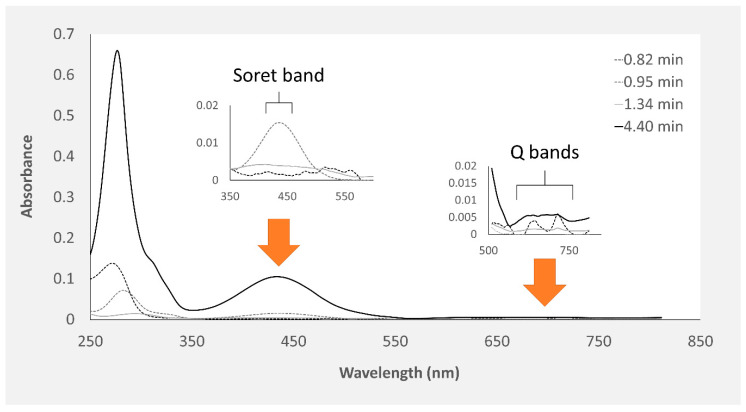
Absorption spectra of the four main pigments in *Eulalia viridis* proboscis crude pigment extracts spectra retrieved from HPLC-DAD analyses (each pigment’s spectrum is identified by its retention time). The Soret (350–500 nm) and Q (580–750 nm) bands typical of porphyrinoids are highlighted.

**Figure 5:**
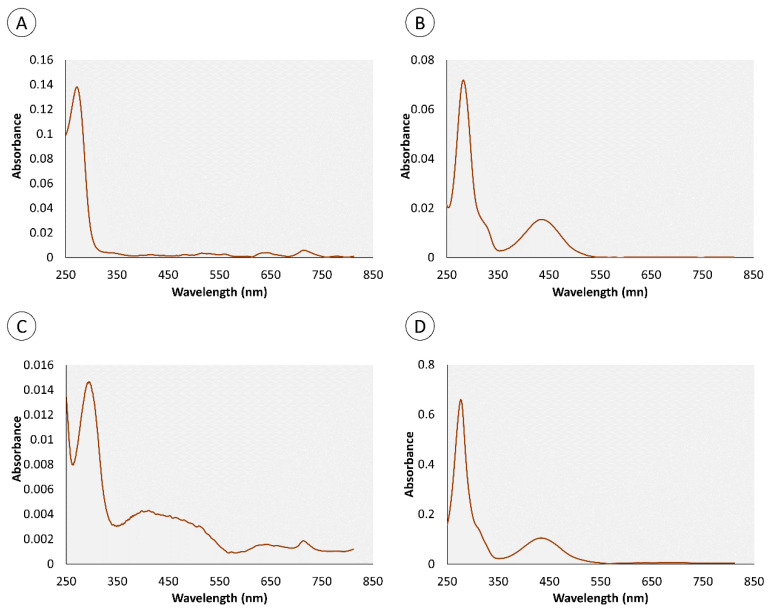
Spectra of the principal pigments in the crude extracts from the proboscis of *Eulalia viridis* fractionated by HPLC-DAD. The proboscis yielded mainly two yellowish and two greenish pigments. (**A**) green pigment (0.82 min); (**B**) yellow pigment (0.95 min); (**C**) green pigment (1.34 min); (**D**) yellow pigment (4.40 min). The pigments detected at retention times of 0.82 and 1.34 min are specific to the proboscis.

The proboscis extract yielded two green and two yellowish main pigments with porphyrinoid signatures (Figure 4). Among the four main compounds, two yellow pigments were detected at retention times of 0.95 and 4.40 min, presenting maxima at both 282 and 286 nm, respectively. To this pigment two greenish pigments detected at retention times of 0.82 and 1.34 min were added, with absorbance maxima at 272 and 257 nm, respectively, which were exclusive to this organ (Figure 5). The yellow pigments detected in the proboscis are similar to those described earlier in the skin, judging from similar retention times (0.95 and 4.40 min) and absorbance magnitudes. As previous, the yellow pigment shown in Figure 5D with the 4.40-min retention time was most abundant, not only in extracts from the proboscis but also comparatively to the skin. Table 1 summarizes the main similarities and differences between pigment extracts from the two organs.

### 2.2. Comet Assay

**Figure 6:**
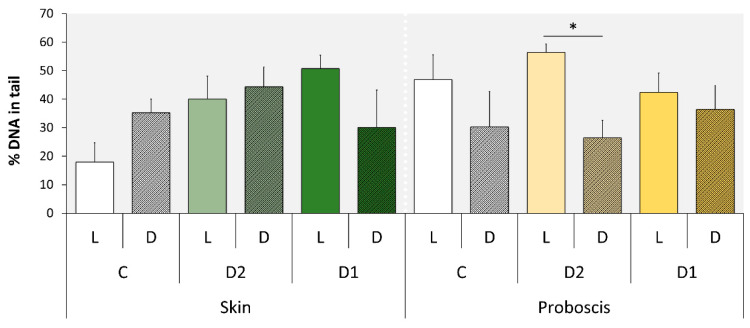
Comet assay results (as % DNA in tail) from the gills of *Mytilus* exposed to two dilutions of pigment extracts corresponding to the nominal concentrations of 100% (D1) and 50% (D2), plus controls, i.e., gills treated with PBS only, which was the vehicle for pigment extracts (C). Mussels were exposed to the extracts from skin and proboscis mixtures under light (L) and dark (D) conditions. The results are expressed as means + SD. [*] indicates significant differences between L and D conditions (*t*-test, *p* < 0.05).

**Figure 7:**
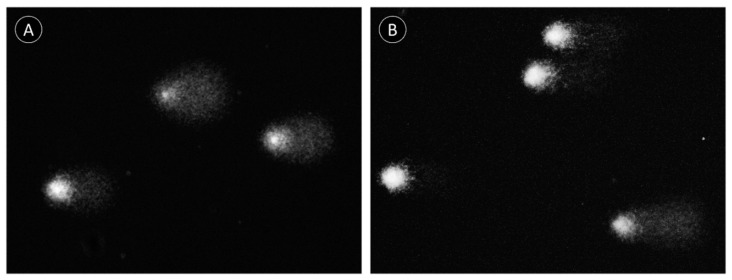
Exemplificative Comet fields from mussel gills treated with pigment extracts from *Eulalia* illustrating the differences between light and dark treatments. (**A**) Gills exposed to 50%-diluted extracts from the proboscis (light treatment); (**B**) same as previous but gills subjected to dark conditions, evidencing lower DNA damage, as seen by higher and lower head and tail intensities, respectively, comparatively to the previous.

The light/dark experimental treatment (here identified as the two-factor variable “treatment”) was the only explanatory variable found to significantly modulate DNA damage (Table 2). In accordance, significant differences were found between the L (light) and D (dark) experimental conditions in gills exposed to extracts from the proboscis, albeit only for the 50% concentration (D2), attaining a maximum of ≈56% DNA in tail (Figure 6). In the case, gills exposed to the extract, in the light, revealed highest damage by ca. two-fold on average, relative to the dark condition (Figure 7). Even though pigment extracts from the skin failed to elicit significant differences between experimental conditions, regardless of dilution, the highest level of DNA damage scored in gills exposed to this extract was recorded in gills subjected to light and exposed to the highest (100%) concentration of pigments (termed D1), attaining an average of 51% DNA in tail.

### 2.3. Daphnia Immobilization Assay

**Figure 8:**
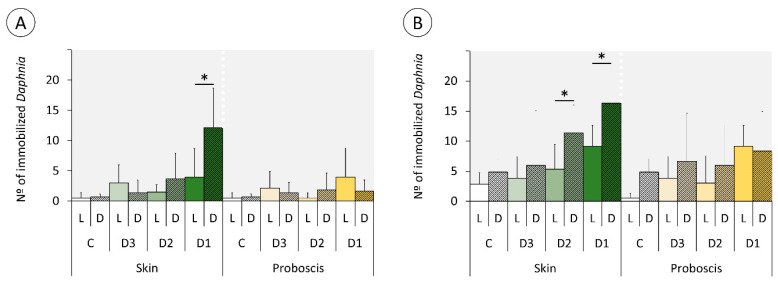
*Daphnia* immobilization assay after exposure to three dilutions of pigment extracts corresponding to the nominal concentrations of 100% (D1), 50% (D2), and 10% (D3), plus controls (C). *Daphnia* were exposed for 1 h to the extracts from skin and proboscis mixtures under light (L) and dark (D) conditions and analyzed after (**A**) 24 h; (**B**) 48 h. The results are expressed as mean immobilized individuals (out of 20) + SD. [*] indicates significant differences between L and D treatment conditions (*t*-test, *p* < 0.05).

All explanatory variables (“treatment”, “exposure time”, “pigment”, and “dilution”) were found to significantly modulate the immobilization of *Daphnia* (Table 3). Extracts from the skin were responsible for the overall highest immobilization rates, which tended to increase with concentration and total exposure time after the initial 1-h light or dark treatment (Figure 8). However, contrarily to the previous findings, exposure to pigment extracts in the dark tended to cause highest effects either at 24 (Figure 8A) or 48 h (Figure 8B), albeit the effects being more significant for assays conducted with the most concentrated extracts from the skin. Accordingly, the highest immobilization rates resulted from *Daphnia* exposed to the most concentrated (D1) skin pigment extract, followed by exposure to D2, both after 48 h, hitherto the dark treatment attaining a ca. two-fold increase relatively to the light condition. Still, after 24 h of the experiment this effect was almost three-fold higher.

## 3. Discussion

In accordance with our previous findings (Martins et al. [20]), the extracts from the “greenish” skin and the “yellowish” proboscis of *Eulalia viridis* contain multiple pigments and sustain very significant inter-organ variation. However, the extraction methods were distinct between the two works, albeit HPLC-DAD being used for fractioning in either case. Whereas we previously used HCl:acetonitrile to extract the pigments, in the present work we opted for extraction in PBS to obtain a physiologically compatible vehicle for the bioassays. The most notorious difference is the higher representativity of individual yellowish pigments in the skin found in the current work relatively to Martins et al. [20]. Nonetheless, the main pigments, in particular the yellow compound retrieved at retention time 4.40 min (Figure 3D and Figure 5D) is seemingly present in the extracts from our preceding work. Moreover, this pigment shows the expected porphyrinoid signature. The methodological differences between the two works are likely responsible for disparities and render direct comparisons difficult, taking into account extraction efficiencies. However, it is clear from the current results that many porphyrinoid pigments in *E. viridis* are hydrophilic and compatible with PBS, which has important implications for biotechnological endeavors. It must be noted, though, that our previous paper already suggested the amphiphilic nature of porphyrinoids from *Eulalia*, similarly to what has already been described for other porphyrin-derived pigments [22]. In addition, the advantages of amphiphilic substances in therapeutics, as they can be transported through the blood stream and have facilitated crossing through the phospholipid by-layer, leads to substantial efforts to produce these compounds synthetically for the purpose of PDT (see for instance Malatesti et al. [22]). The discovery of natural bioproducts with these properties, as in the present case, is, therefore, an important biotechnological asset.

The two very distinct toxicity-testing procedures yielded divergent, albeit complementary results, in any case the presence or absence of light being a significant factor. The duration of the assays (i.e., total time of exposure) is inferred to be a key to explain the differences. Indeed, the shorter-term (1 h) assays with mussel gills revealed a trend for higher DNA-damage effects under light, whereas the *Daphnia* immobilization assay yielded the opposite tendency after 24 and 48 h of exposure following the initial 1 h treatment under light or dark conditions. Consequently, despite the differences between model and endpoint, time of exposure is seemingly a major player in the modulation of toxicity because the generation of singlet oxygen by photodynamic substances occurs swiftly after irradiation with visible light [23]. Evidently, this depends on oxygen supply, which is certainly more reduced in the test medium of *Daphnia*, especially after 48 h, even assuming minimal degradation of the pigments in the test medium or by the cladocerans’ own mechanisms of porphyrinoid catabolism and elimination. Consequently, the photodynamic effect of porphyrinoids was more evident in DNA damage determined by the standard alkaline Comet assay, which is directly sensitive to singlet oxygen radicals in the short term, with particular respect to the formation of oxidized purines [24,25]. On the other hand, after 24 and 48 h of exposure, low oxygen and photosensitizer exhaustion seemingly reduced the photodynamic effects of pigments, leaving dark toxicity of phorphyrinoids as the most evident effect. This effect has already been described for a range of porphyrin-based photosensitizers in vitro [25,26]. Even though dark toxicity for these compounds in vivo is not well understood, the results suggest significant effects in *Daphnia*. However, details on the toxicity, metabolism, and elimination of heme and its secondary metabolites are scarce (refer, for instance, to Lamkemeyer et al. [27] and references therein on *Daphnia* hemoglobin). Still, Fabris et al. [28] found *Daphnia magna* particularly sensitive to both light and dark toxicity of certain porphyrinoids after short term (1 h) and 24–48 h of incubation. It is noteworthy that the same authors employed the standard *Daphnia* immobilization assay as well. These results are thus in good alignment with the current study and show that the toxicodynamics and toxicokinetics of photosensitizers is complex as it depends on the ratio between light:dark toxicity and how it changes with time. Even though the sensitivity of our models and endpoints cannot be directly compared, the findings also demonstrate the importance of testing both immediate and longer-term effects of exposure to isolate light and dark toxicity of porphyrinoids. This subject is of particular relevance for further studies to investigate the potential of pigments in PDT, as dark toxicity can cause significant effects during the period of elimination, after the effective photodynamic capability of pigments has been exhausted.

There are also very noticeable differences between the effects caused by the two extracts that ultimately enable shortlisting the pigments with the most promising properties as photosensitizers. The findings show that the extracts from the proboscis, where the yellow pigment with 4.40 min retention time is by far more abundant than in the skin (which makes it responsible for the difference in color between the two extracts), hold higher phototoxicity. This can be ascertained from the comparison between Figure 6 and Figure 8. Most likely, the majority of the phototoxicity causing the increment of DNA damage relatively to the dark treatment shown in Figure 6 is due to this same yellow pigment, despite inconclusive dose-response effects. On the other hand, the mixture of pigments in the skin extracts, which includes the same compounds albeit in much lower proportion, caused higher dark toxicity. Altogether, the results indicate that this yellow pigment is an interesting candidate for further research as an effective photosensitizer. It must be noted, though, that the lack of a clear dose-response is in part due to significant inter-replica variation and reduced number of concentrations tested. Nonetheless, we must emphasize that at this stage we were testing crude extracts and not the purified pigments, which also contributed to the overall variation. Further toxicity testing involving the isolation of this candidate pigment are needed, as well a complete chemical characterization. It must also be noticed that the extracts from the skin caused DNA damage and suggest a potential dose-response and light-mediated toxicity as well. However, as pointed out in our previous work, this organ holds a much wider variety of pigments than the proboscis [20], which complicates, at this stage, isolating specific substances of interest and obtaining sufficient amounts for analyses.

It can be inferred that the light toxicity of porphyrins is certain to cause deleterious effects to the worm itself. This consequence has been noted before and has, inclusively, been suggested as one of the reasons why Polychaeta are so difficult to rear in captivity [29], which explains the need to add shelter and provide dimmed light to *Eulalia* in the laboratory [18]. Considering that *Eulalia* is a diurnal forager of the rocky intertidal, its pigments likely have two important roles. First, they are either toxic or repellent to predators and parasites that cause rupture to the pigment cells where the pigments are “safely” stored in granules [19,20]. Second, they warn of excessive exposure to daylight by causing physiological stress, which can be considered a form of non-nervous sensorial arrangement. The fact that *Eulalia* uses its proboscis as the main organ for sensing and preying [19,21] explains the natural difference between the pigmentation of the skin as a major adaptive trait. These particular features, which are probably not circumscribed to *Eulalia* or even to the Order Phyllodocida, show that the Polychaeta are very promising targets for the bioprospecting of photoactive tetrapyrrolic pigments.

In conclusion, the present work revealed different porphyrin-like pigments in the body of the Polychaeta *Eulalia*, distributed differentially between the proboscis and the skin. The differential pigmentation pattern between the two organs is also reflected in distinct light:dark toxicity ratios that are influenced by time of exposure, as light-dependent toxicity occurs swiftly whereas light-independent will occur in a longer timeframe until the substances are metabolized and finally eliminated from the recipient organism. With this respect, the two bioassay procedures with mussel gills and *Daphnia* (1 and 48 h, respectively), showed the importance of performing short and longer tests to evaluate the unique toxicodynamics of photosensitizers. From the aggregate findings we were able to detect a specific pigment, yellowish in color, present in both organs albeit much more concentrated in the proboscis of the worm. This pigment, in particular, is a serious candidate as photosensitizer: (i) it is likely amphiphilic and compliant with PBS, which is an advantage for distribution during therapeutics; (ii) its light toxicity is considerably higher than its dark toxicity, and (iii) its reduced toxicity with time in vivo may be an indicator of rapid exhaustion and facilitated elimination. These highly promising results also confirm that marine invertebrates are a prolific source of natural porphyrinoids, a class of substances with high value for therapeutics and whose research is still mostly based on synthetics.

## 4. Materials and Methods

### 4.1. Animals

Adult *E. viridis* (ca. 5–10 cm length) were collected by hand in Parede Beach, an intertidal rocky beach in Western Portugal (38°41′42″ N; 09°21′36″ W), during the low tide. Organisms were then transported to the laboratory and kept in a mesocosm recreating their natural habitat consisting of dark-walled aquaria, equipped with a system providing constant aeration and water recirculation, to which natural rocks were added, with barnacles and mussels collected from the same area to provide shelter and feed to the worms, as developed by Rodrigo et al. [18]. Salinity, water temperature and photoperiod were maintained restrained at about 30, 18 °C and 16:8 h light-dark, respectively. Animals were thus acclimatized for 7–14 days until pigment extraction.

Mussels (*Mytilus* sp.) were also collected as models for the ex vivo assay. Laboratory cultures of wild-type *Daphnia pulex* were maintained in an aquarium with filtered pond water, replicating optimal environmental conditions for their reproduction (temperature: 20 ± 2 °C; pH: 7.5; photoperiod: 16 h light:8 h dark). *Daphnia* were fed with a mixture of *Arthrospira platensis* and *Saccharomyces cerevisiae*, following Pellegri et al. [30] and Santos et al. [31].

### 4.2. Pigment Extraction

Worms were immobilized by hypotonic shock and microdissected to collect proboscis and skin (factually the body wall containing epidermis plus underlying musculature). The organs were then homogenized separately with a pestle in cold Dulbecco’s phosphate-buffered saline (PBS), pH 7.5 to extract the hydrophilic fraction of pigments. Samples were afterwards centrifuged for 5 min, 5000 *g* at 4 °C. The supernatant containing the pigments was collected and immediately placed on ice and in the dark. The pellet was subjected to repeated extractions until the supernatant was visibly devoid of pigments. The supernatants were then pooled. Each pooled sample contained extracts from approximately five animals.

The crude extracts of either organ were then analyzed spectrophotometrically at 440 and 700 nm, the maxima described by Martins et al. [20]. Extracts were then diluted in PBS, with the absorbance at 440 being used to normalize the dilutions until samples yielded an absorbance value of 1 (D1), corresponding to the 100% concentrated extract. This solution was necessary to overcome the absence of suitable standards for the mixtures of pigments in the extracts from either organ. Serial dilutions D1 (100%) were then produced, termed D2 and D3, corresponding to 50% and 10% of D1, respectively. Pigment extraction was performed in a dim-lighted environment to avoid photodegradation of pigments. Exposure of worm tissues and extracts to ambient light and air was kept to the minimum. Analysis and toxicity testing involved only fresh extracts.

### 4.3. Chemical Characterization of Pigments

Crude extracts of the two organs (skin and proboscis) were filtered with a GHP filter before analysis. Pigments were analyzed based on the protocol for separation and quantification of human bilins developed by Woods and Simmonds [32], with many optimizations. In brief, high-performance liquid chromatography (HPLC) analyses were conducted on a Merck-Hitachi instrument equipped with a diode array detector (DAD), scan range 200–800 nm (Merck-Hitachi L-4500 Diode Array Detector, Merck, Poole, UK), operating at 20 °C, using a reversed-phase analytical column (RP-HPLC, Onix^®^ Monolithic C18 column, 100 × 4.6 mm i.d., 13 nm and 2 μm). Samples were prepared in MeOH and the injection volume was 20 μL. Preliminary assays showed that optimal peak separation was obtained with sodium phosphate buffer 10 mM, pH 3.5 (solvent A) and pure MeOH (solvent B) at a flow rate of 2 mL/min: linear gradient from 45% to 95% B for 10 min and 95% B for 2 min. The total run time excluding equilibration was 12 min. Linear gradient from 30% to 60% B for 5 min and linear gradient from 60% to 95% B for 10 min. The total run time excluding equilibration was 15 min. Throughout analysis, the pressure was maintained at about 67 bar and the temperature column kept at environmental temperature (20 ± 2 °C).

### 4.4. Experimental Design

#### 4.4.1. Assays with *Mytilus* sp. Gills

Ex vivo bioassays using *Mytilus* sp. gills were performed to investigate DNA damage resulting from exposure to pigments under light or dark conditions. For the purpose, valves of live mussels (ca. 40 mm in length) were carefully separated to retain integrity of gills and the visceral mass swiftly removed. Assays began immediately after excision. One valve being used as a test sample (pigment extract, diluted in PBS as described above) and the other as its respective control (PBS only). For both crude extracts, proboscis (P) and skin (S), the test valve was exposed to 1 mL of D1 or D2 dilutions, while the control was treated with 1 mL of PBS. Test valves and controls were subjected to either ambient light (L), i.e., indirect daylight, or dark (D) conditions for 1 h and processed immediately afterwards. All assays were made in triplicate (*n* = 3).

#### 4.4.2. Comet Assay

Damage to DNA was evaluated using an adaptation of the alkaline single-cell gel electrophoresis (Comet) assay, developed by Singh et al. [33], adapted by Raimundo et al. [34] for molluscan solid tissue. Freshly harvested gill samples were minced with pliers in 700 mL of cold PBS and centrifuged for 1 min at 1200 rpm. The supernatant (clear cell suspension) was diluted in 1% *m*/*v* molten (37–40 °C) low melting point agarose (LMPA) prepared in PBS. Afterwards, two 80-μL drops of LMPA cell suspensions were placed on slides pre-coated with 1.2% *m*/*v* of normal melting point agarose (dried for at least 48 h) and covered with a coverslip. After LMPA solidification (15 min, 4 °C) coverslips were removed and the slides immersed in cold lysis buffer (0.45 M NaCl (*m*/*v*); 40 mM EDTA (*m*/*v*); 5 mM Tris pH 10) for 1 h. Slides were then placed in cold electrophoresis buffer (0.1 mM EDTA; 0.3 M NaOH, pH 13) for 40 min to allow DNA-unwinding and expression of alkali-labile sites. Electrophoresis was run at 25 V for 30 min at 4 °C. Then, the slides were neutralized in 0.2 M Tris-HCl, pH 7.5, and dried with methanol for archiving before analysis. Rehydrated slides were stained with GreenSafe (Nzytech, Portugal) [35] and analyzed with a DM 2500 LED microscope adapted for epifluorescence with an EL 6000-light source (Leica Microsystems). Scoring was done with CometScore 1.6 (Tritek), with 100 nucleoids being analyzed per slide. The percentage of DNA in tail was considered as a direct measure of DNA damage [36].

#### 4.4.3. Acute Toxicity Assay with *Daphnia pulex*

Based on standard guidelines for toxicity testing with *Daphnia* sp. [37,38,39], daphnids with less than 24 h were selected for the assay. For the purpose, several females with mature eggs in the brood pouch were transferred to a 90-mm Petri dish containing with distilled water 12 h before the experiments. Hatched juveniles (daphnids) were afterwards collected and used for the tests. The test replicate consisted of a well of a 24-well clear bottom microplate (2-mL well) containing 20 daphnids. Each well contained 0.2 mL of extract and 1.8 mL of distilled water. Plates were then exposed under dark (D) of light (L) conditions, for 1 h, to dilutions D1, D2, or D3 of P and S pigment extracts, plus controls, which consisted of adding 0.2 mL of PBS to the test water (*n* = 6) and afterwards incubated in unchanged medium, in the dark, until analysis. The number of immobilized daphnids was evaluated after 24 and 48 h from the beginning of the exposure.

### 4.5. Statistical Analysis

Homoscedasticity of data was assessed by the Levene’s test. After invalidation of this assumption for parametric analysis, we used generalized linear models (GLM) through a Poisson regression with a log link function, considering dilution, light/dark treatment, organ, and time (duration) of the experiment (in the case of the *Daphnia* assay) as explanatory variables. Analysis of variance based on deviance was carried out to assess the effect of independent variables on DNA damage and immobilization of *Daphnia*. Complementary comparisons were done with Student’s *t*-test. All analyses were done using R [40]. Generalized linear models were computed with the package *glm2*.

## Figures and Tables

**Table 1:** Summary of the main pigments in PBS extracts from the skin and proboscis of *Eulalia viridis*, based on data retrieved from HPLC-DAD.

Retention Time (min)	Organ		Color		Absorbance Maximum (nm)	
	Skin	Proboscis	Green	Yellow	Skin	Proboscis
0.95	•	•		•	280 nm	282 nm
4.4	•	•		•	276 nm	286 nm
0.82	–	•	•		–	272 nm
1.34	–	•	•		–	257 nm
1.8	•	–		•	288 nm	–
3.43	•	–	•		288 nm	–

[•] and [–] indicate presence or absence, respectively.

**Table 2:** Results from ANOVA for GLM (based on sequential analysis of deviance) for the Comet assay on mussel gills exposed to pigments.

Variable	df	Deviance Residuals	df	Residual Deviance	*p*
Treatment	1	0.44403	46	5.0954	0.02629 ^1^
Pigment	1	0.20322	45	4.8922	0.13280
Dilution	2	0.13029	43	4.7619	0.48465

^1^ significant with *p* < 0.05.

**Table 3:** Results from ANOVA for GLM (based on sequential analysis of deviance) for the *Daphnia* immobilization assay.

Variable	df	Deviance Residuals	df	Residual Deviance	*p*
Treatment	1	36.461	190	862.43	1.558 × 10^−9 1^
Pigment	1	31.388	189	795.04	2.113 × 10^−8 1^
Exposure time	1	143.122	188	651.92	<2 × 10^−16^ ^1^
Dilution	3	160.977	185	490.94	<2 × 10^−16^ ^1^

^1^ significant with *p* < 0.01.
